# A Novel Path to Obesity

**DOI:** 10.1371/journal.pbio.1001507

**Published:** 2013-03-12

**Authors:** Caitlin Sedwick

**Affiliations:** Freelance Science Writer, San Diego, California, United States of America

The hormone leptin is critical for maintaining metabolic homeostasis. Secreted by adipose tissue after a meal, leptin binds to specific leptin receptors in certain brain regions, particularly the arcuate nucleus (Arc) region of the hypothalamus. Upon receptor binding, it stimulates phosphorylation of signaling proteins such as Jak and Stat3. These in turn activate the production of neurotransmitters that control neuronal circuits dedicated to inhibiting food intake and regulating energy expenditure. Tellingly, loss of leptin, or of leptin receptors, causes obesity due to impaired satiety control and disrupted energy balance. But, as Viola Nordström, Richard Jennemann, Hermann-Josef Gröne, and colleagues demonstrate in this week's *PLOS Biology*, there are other ways leptin signaling can become disrupted.

**Figure:**
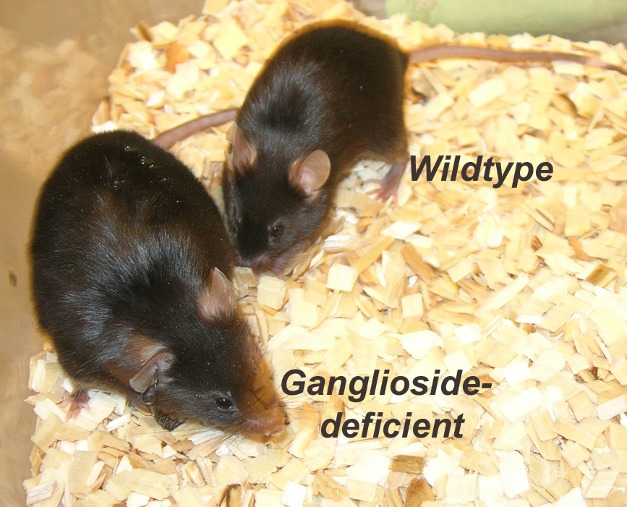
The function of membrane receptors regulating feeding and fat burning depends on gangliosides surrounding them. Mice lacking gangliosides in hypothalamic neurons develop obesity.

Nordström et al. were interested in glycosylceramide synthase (GCS), an enzyme that controls production of many lipids, including glycosphingolipids and gangliosides (which are particularly concentrated in cell membranes). The researchers' attention was first drawn to this protein because certain gangliosides are known to be important for proper signaling by the insulin receptor, which is crucial for metabolic balance. They were curious whether GCS and the lipids it produces might be important for regulation of brain metabolic pathways, but loss of GCS in all neurons is known to be lethal, making it difficult to study. So, in order to study the importance of GCS in neurons, the authors created a genetically modified mouse in which GCS expression can be specifically knocked out in only certain regions of the adult forebrain, including the hypothalamic Arc.

In these mutant mice, GCS expression is only lost once the animals are injected with a specific chemical trigger. Before trigger injection, Nordström and colleagues observed that the mice appear normal. But three weeks after the trigger is injected, the animals begin accumulating white adipose tissue, eventually becoming obese. The researchers observed that these animals have an early tendency to eat slightly more than wild type animals, which could explain the animals' initial fat accumulation. Their overeating stops later in life, but their energy use becomes so disrupted that they become chronically hypothermic and also develop an inability to use fat as an energy source, so they remain obese. However, when GCS expression was reconstituted specifically in the Arc through local injection of viruses carrying a functional copy of the GCS gene, the animals gained significantly less weight. This suggests that GCS loss specifically in the Arc (as opposed to other areas of the brain) plays an important role in causing the observed metabolic imbalances.

This discovery prompted the authors to examine why loss of GCS in the Arc has these effects. First, they explored whether GCS loss impairs the overall viability or function of neurons. Their experiments showed that GCS-deficient Arc neurons die at the same rate, have similar physical structure to, and can conduct nerve impulses like normal neurons do. Similarly, they found no major functional defects when a cultured hypothalamic cell line (called N41) was treated with a drug that depletes gangliosides from the membrane. These studies ruled out the possibility that GCS loss causes gross impairment of neuron viability or functionality.

Because leptin signaling in the Arc is known to be important for metabolic homeostasis, Nordström et al. next decided to investigate whether leptin signaling is disrupted in the absence of GCS. Indeed, immunofluorescence studies of slices from Arc tissue showed that in the absence of GCS, leptin treatment fails to stimulate the expected appearance of phosphorylated signaling proteins in neurons. The impaired signaling response to leptin was accompanied by a simultaneous increase in the expression of neurotransmitters that enhance food intake. Leptin insensitivity of GCS-deficient Arc neurons may therefore explain the animals' initial overeating behavior and their decreased metabolic activity.

These findings led the authors to wonder how GCS loss might impair Arc neurons' response to leptin. Because gangliosides are known to directly associate with and assist signaling by other types of receptors, Nordström et al. theorized that gangliosides might be required for the leptin receptor to function. This idea was supported by in vitro experiments showing that major membrane gangliosides associate with the leptin receptor in N41 cells, and that this association increases upon leptin stimulation. Furthermore, they found that ganglioside depletion from N41 cells also impairs leptin receptor signaling after leptin stimulation.

Taken together, these data indicate that loss of membrane gangliosides (likely caused by loss of GCS) impairs the cells' ability to respond to stimulation by leptin. By targeting ganglioside expression in the hypothalamic arcuate nucleus, Nordström and colleagues disrupted metabolic regulation in their mice. Might alterations to hypothalamic membrane gangliosides also affect human leptin signaling and metabolic balance? Further investigation into this novel idea is needed.

**Nordström V, Willershäuser M, Herzer S, Rozman J, von Bohlen und Halbach O, Meldner S, et al. (2013) Neuronal Expression of Glucosylceramide Synthase in Central Nervous System Regulates Body Weight and Energy Homeostasis. doi:10.1371/journal.pbio.1001506**

